# Characteristics and costs in adults with acute poisoning admitted to the emergency department of a university hospital in Belgium

**DOI:** 10.1371/journal.pone.0223479

**Published:** 2019-10-04

**Authors:** Anne-Marie K. Descamps, Dominique M. Vandijck, Walter A. Buylaert, Martine A. Mostin, Peter De Paepe

**Affiliations:** 1 Antigifcentrum/Centre Antipoisons, Brussels, Belgium; 2 Department of Public Health of Ghent University, Ghent, Belgium; 3 Department of Medicine and Health Sciences of Hasselt University, Diepenbeek, Belgium; 4 Department of Emergency Medicine of Ghent University Hospital, Ghent, Belgium; Osakidetza Basque Health Service, SPAIN

## Abstract

**Objective:**

The aims of this study were to assess the characteristics of all acute poisoning admissions among adult emergency department (ED) patients, to identify factors associated with admission and to calculate direct medical cost.

**Methods:**

Data of 2017 (1^st^ January to 31^st^ December) were collected and analyzed retrospectively using patients’ medical records and hospital invoices. Factors associated with type of hospitalization were identified using appropriate statistics.

**Results:**

A total of 1,214 hospital admissions were included, accounting for 3.6% of all ED admissions. Men (62.2%) and the age group 21–40 years (43.0%) accounted for the largest proportion. Substances most commonly involved were ethanol (52.9%), benzodiazepines (9.7%), cocaine (4.9%), cannabis (4.6%), antidepressants (4.6%) and psychostimulants (4.6%). A total of 4,561 treatment acts were recorded, most commonly monitoring of vital signs (63.6%) and medication and/or intravenous drip administration (62.9%). Patients were discharged home after having received care in the emergency department (ED-amb) in 54.5% of admissions, were admitted to the emergency-department-24-hours-observation unit (ED-24h) or were hospitalized (Hosp) in 24.6% and 20.9% of admissions, respectively. Factors found to be associated with hospitalization type were age, hour of admission, victim location, degree of severity, use of antidotes, involvement of antidepressants, antipsychotics, psychostimulants, benzodiazepines and ethanol. Total cost was €1,512,346 with an average of €1,287 per admission.

**Conclusion:**

Poisonings entail a considerable percentage of patients admitted to an ED and financial burden. In particular, ethanol poisonings account for the largest proportion of all ED admissions. Comparison of our figures with other data is hampered by the heterogeneity in inclusion criteria. Availability of a uniform template would facilitate comparison and allow better monitoring policies for prevention and cost reduction.

## Introduction

Poisoning poses a significant global public health problem. According to WHO data, an estimated 193,460 people die worldwide from unintentional poisoning [1]. Hospitals, and in particular emergency departments (ED), are faced with a considerable number of admissions leading to a substantial number of hospitalizations and costs.

Studies on the characteristics of acute poisonings have been conducted in a number of countries. Some of them focused on cases in EDs [2–12], while others on cases in hospitals [13–17]. They give an idea of the demographic characteristics of the patients, the time of admission, the substances involved, the therapeutic measures taken and the outcome of the patients. With regard to cost studies, some include only ED-costs [18–21], while others also hospitalization costs [22] or ICU costs [23,24]. Other studies are limited to the financial burden of poisoning by drugs, medicaments and biological substances [25], illicit drugs [26], opioids [27], alcohol and drug overdoses [28] or self-poisoning cases [29–31].

In Belgium, studies of acute poisonings are scarce and limited to the analysis of cases treated in the ED [32] or focus on alcohol intoxications [19] or deliberate self-poisonings [20].

The monitoring of poisoning trends and costs is important to evaluate the appropriateness and quality of care, to identify factors associated with the type of hospitalization and to give an idea of the costs involved. In this context, it may also be clear that the development of a uniform data reporting tool would facilitate comparison of studies. Therefore, the aims of the present study are (1) to inventarize the characteristics of acute poisoning admissions to the ED of a Belgian university hospital, (2) to identify risk factors for hospitalization type and (3) to calculate general direct medical costs of acute poisonings.

## Materials and methods

We used the “Strengthening the Reporting of Observational Studies in Epidemiology Statement” STROBE as a guideline for reporting [33].

### Study design and setting

This study is a retrospective analysis of data considering patient records of all poisoning-related admissions of patients aged 14 years or older admitted to the ED of the Ghent University Hospital (GUH). GUH is a 1,062-beds tertiary care referral center in Belgium with about 34,000 ED admissions per year and where more severe cases are admitted. It is serving an urban area with many students and covering to a lesser extent a rural area. This should be kept in mind when comparing our results with other studies. Data were collected from 1 January 2017 to 31 December 2017.

### Inclusion criteria

To avoid overlooking patients who came in with a different chief complaint but were also poisoned, all ED patients with the codes for intoxication, carbon monoxide intoxication, suicide attempt, social, mental or psychological reason, were screened for poisoning. They were included when the reason for admission could be encoded in T36-T50 (poisoning by drugs, medicaments and biological substances) or in T51-T65 (toxic effects of substances chiefly nonmedicinal as to source) of the International Classification of Diseases (ICD-10) [34]. For each admission included, the first author (AMD) and another researcher (KL) considered independently if inclusion was justified. The cases without agreement were discussed with the department head of GUHED (PDP) and were included after consensus.

### Variables

All admitted patients were triaged according to the Manchester Triage Scale [35]. After treatment, ambulatory patients (ED-amb) were discharged home, while patients requiring observation were admitted to the ED-24-hours-observation-unit (ED-24h). Some patients had to be admitted to the hospital ward (Hosp) or transferred to the intensive care unit (ICU) for further monitoring.

The cases were categorized according to (1) accidental (unintentional) poisoning, (2) use of substances of abuse, (3) intentional self-harm or (4) undetermined cause of poisoning. Poisoning was considered as accidental in case of “external causes of poisoning and accidents (e.g. taking the wrong medication) with the agent taken for neither self-harm nor intoxication purposes” [17]. A substance of abuse was defined as “recreational use of substance of abuse” [36]. Intentional self-harm was defined as “purposely self-inflicted poisoning”, as categorised in ICD-10, X60-X69 [34]. The term undetermined poisoning was used when the underlying reason was unclear.

Tables 1 to 3 present the analysis of the characteristics and agents for all admissions, including those of patients readmitted during the study period. Since some patients were admitted more than once, we performed also a separate analysis in which readmissions were accounted for by considering all patients who presented only once as well as patients with readmissions; for the latter only their first admission was taken into account. These data are shown in the tables in S1–S5 Tables.

**Table 1:** Demographic data and characteristics of admissions for poisoning to the Ghent University Hospital according to hospitalization type.

	Total	ED-ambulatory care	ED-24-hours-observation	Hospitalization/ICU	p-value^1^
	1,214	661	299	254	
	n (%)	n (%)	n (%)	n (%)	
**Gender**					0.017
Male	755 (62.2)	435 (65.8)	171 (57.2)	149 (58.7)	
Female	459 (37.8)	226 (34.2)	128 (42.8)	105 (41.3)	
*Total*	*1*,*214 (100*.*0)*	*661 (100*.*0)*	*299 (100*.*0)*	*254 (100*.*0)*	
**Age**					<0.001
14-20y	213 (17.5)	169 (25.6)	29 (9.7)	15 (5.9)	
21-40y	522 (43.0)	295 (44.6)	119 (39.8)	108 (42.5)	
41-60y	387 (31.9)	161 (24.4)	118 (39.5)	108 (42.5)	
>60y	92 (7.6)	36 (5.4)	33 (11.0)	23 (9.1)	
*Total*	*1*,*214 (100*.*0)*	*661 (100*.*0)*	*299 (100*.*0)*	*254 (100*.*0)*	
**Marital status**					0.099
Unmarried/widow(er)/divorced	834 (83.6)	417 (86.2)	223 (81.1)	194 (81.2)	
Married/cohabiting	164 (16.4)	67 (13.8)	52 (18.9)	45 (18.8)	
*Total*	*998 (100*.*0)*	*484 (100*.*0)*	*275 (100*.*0)*	*239 (100*.*0)*	
**Residence**					0.63
Ghent	475 (39.1)	253 (38.3)	124 (41.5)	98 (38.6)	
Outside Ghent	739 (60.9)	408 (61.7)	175 (58.5)	156 (61.4)	
*Total*	*1*,*214 (100*.*0)*	*661 (100*.*0)*	*299 (100*.*0)*	*254 (100*.*0)*	
**Day of the week admission**					<0.001
Monday	141 (11.6)	60 (9.1)	42 (14.0)	39 (15.4)	
Tuesday	171 (14.1)	85 (12.9)	54 (18.1)	32 (12.6)	
Wednesday	143 (11.8)	78 (11.8)	32 (10.7)	33 (13.0)	
Thursday	159 (13.1)	73 (11.0)	55 (18.4)	31 (12.2)	
Friday	199 (16.4)	124 (18.8)	31 (10.4)	44 (17.3)	
Saturday	194 (16.0)	121 (18.3)	38 (12.7)	35 (13.8)	
Sunday	207 (17.1)	120 (18.2)	47 (15.7)	40 (15.7)	
*Total*	*1*,*214 (100*.*0)*	*661 (100*.*0)*	*299 (100*.*0)*	*254 (100*.*0)*	
**Time of admission**					<0.001
8am-12am	103 (8.5)	50 (7.6)	20 (6.7)	33 (13.0)	
12am-4pm	176 (14.5)	69 (10.4)	45 (15.1)	62 (24.4)	
4pm-8pm	245 (20.2)	96 (14.5)	86 (28.8)	63 (24.8)	
8pm-12pm	275 (22.7)	103 (15.6)	111 (37.1)	61 (24.0)	
12pm-4am	272 (22.4)	226 (34.2)	23 (7.7)	23 (9.1)	
4am-8am	143 (11.8)	117 (17.7)	14 (4.7)	12 (4.7)	
*Total*	*1*,*214 (100*.*0)*	*661 (100*.*0)*	*299 (100*.*0)*	*254 (100*.*0)*	
**Victim location**					<0.001
Home	727 (59.9)	323 (48.9)	200 (66.9)	204 (80.3)	
Public place	322 (26.5)	243 (36.8)	48 (16.1)	31 (12.2)	
Other	165 (13.6)	95 (14.4)	51 (17.1)	19 (7.5)	
*Total*	*1*,*214 (100*.*0)*	*661 (100*.*0)*	*299 (100*.*0)*	*254 (100*.*0)*	
**Referred by**					<0.001
On its own initiative	657 (54.1)	357 (54.0)	153 (51.2)	147 (57.9)	
Externals, no patient participation	472 (38.9)	273(41.3)	126 (42.1)	73 (28.7)	
General practitioner/physician	77 (6.3)	26 (3.9)	18 (6.0)	33 (13.0)	
Other	8 (0.7)	5 (0.8)	2 (0.7)	1 (0.4)	
*Total*	*1*,*214 (100*.*0)*	*661 (100*.*0)*	*299 (100*.*0)*	*254 (100*.*0)*	
**Transport**					<0.001
By own means	357 (29.4)	167 (25.3)	79 (26.4)	111 (43.7)	
Ambulance	659 (54.3)	386 (58.4)	171 (57.2)	102 (40.2)	
Mobile Intensive Care Unit	198 (16.3)	108 (16.3)	49 (16.4)	41 (16.1)	
*Total*	*1*,*214 (100*.*0)*	*661 (100*.*0)*	*299 (100*.*0)*	*254 (100*.*0)*	

^1^ Chi-square and Fisher’s exact test

**Table 2:** Characteristics, examinations and treatment of admissions for poisoning to the Ghent University Hospital in 2017 according to hospitalization.

	Total	ED-ambulatory care	ED-24-hours-observation	Hospitalization/ICU	p-value^1^
	1,214	661	299	254	
	n (%)	n (%)	n (%)	n (%)	
**Symptoms on admission**					
Changes in consciousness	257 (21.2)	153 (23.1)	57 (19.1)	47 (18.5)	-
Behavioural/ emotional disorders	204 (16.8)	92 (13.9)	57 (19.1)	55 (21.7)	
Other	144 (11.9)	81 (12.3)	36 (12.0)	27 (10.6)	
Non-specific symptoms	131 (10.8)	89 (13.5)	20 (6.7)	22 (8.7)	
Nausea, vomiting	70 (5.8)	26 (3.9)	20 (6.7)	24 (9.4)	
General malaise	58 (4.8)	27 (4.1)	17 (5.7)	14 (5.5)	
Headache	22 (1.8)	11 (1.7)	9 (3.0)	2 (0.8)	
Wounds, swelling, fracture	20 (1.6)	10 (1.5)	7 (2.3)	3 (1.2)	
External signs of bleeding or bleeding	11 (0.9)	8 (1.2)	3 (1.0)	0 (0.0)	
Dermatological, ophtalmological, nose throat ear problems	8 (0.7)	5 (0.8)	2 (0.7)	1 (0.4)	
Signs of neurological failure	6 (0.5)	0 (0.0)	2 (0.7)	4 (1.6)	
Abdominal pain	5 (0.4)	4 (0.6)	1 (0.3)	0 (0.0)	
Retrosternal and thoracic pain	3 (0.2)	1 (0.2)	2 (0.7)	0 (0.0)	
Pain in limbs, neck, shoulder, pelvic region	3 (0.2)	2 (0.3)	0 (0.0)	1 (0.4)	
Palpitations	3 (0.2)	1 (0.2)	1 (0.3)	1 (0.4)	
Dizziness and syncopal feeling	3 (0.2)	2 (0.3)	1 (0.3)	0 (0.0)	
Tremor, coordination disorders	3 (0.2)	0 (0.0)	0 (0.0)	3 (1.2)	
Respiratory problems	2 (0.2)	2 (0.3)	0 (0.0)	0 (0.0)	
Fever and convulsion	1 (0.1)	1 (0.2)	0 (0.0)	0 (0.0)	
Diarrhoea	1 (0.1)	1 (0.2)	0 (0.0)	0 (0.0)	
Unknown	259 (21.3)	145 (21.9)	64 (21.4)	50 (19.7)	
*Total*	*1*,*214 (100*.*0)*	*661 (100*.*0)*	*299 (100*.*0)*	*254 (100*.*0)*	
**Route of exposure**					0.024
Oral/oromucosal	1,047 (86.2)	566 (85.6)	264 (88.3)	217 (85.4)	
Inhalation	65 (5.4)	45 (6.8)	9 (3.0)	11 (4.3)	
>1 way	91 (7.5)	48 (7.3)	23 (7.7)	20 (7.9)	
Other or unknown	11 (0.9)	2 (0.3)	3 (1.0)	6 (2.4)	
*Total*	*1*,*214 (100*.*0)*	*661 (100*.*0)*	*299 (100*.*0)*	*254 (100*.*0)*	
**Manchester Triage Score**					<0.001
Not urgent	222 (18.3)	156 (23.6)	36 (12.0)	30 (11.8)	
Less urgent	748 (61.6)	409 (61.9)	195 (65.2)	144 (56.7)	
(Very) urgent evaluation	226 (18.6)	88 (13.3)	64 (21.4)	74 (29.1)	
Unknown	18 (1.5)	8 (1.2)	4 (1.3)	6 (2.4)	
*Total*	*1*,*214 (100*.*0)*	*661 (100*.*0)*	*299 (100*.*0)*	*254 (100*.*0)*	
**Glasgow coma score**					0.003
<8	30 (2.5)	19 (2.9)	2 (0.7)	9 (3.5)	
8–14	265 (21.8)	141 (21.3)	79 (26.4)	45 (17.7)	
15	644 (53.0)	363 (54.9)	157 (52.5)	124 (48.8)	
Unknown	275 (22.7)	138 (20.9)	61 (20.4)	76 (29.9)	
*Total*	*1*,*214 (100*.*0)*	*661 (100*.*0)*	*299 (100*.*0)*	*254 (100*.*0)*	
**Number of days hospitalization**					<0.001
0	661 (54.4)	662 (100.0)	0 (0.0)	0 (0.0)	
1	313 (25.8)	0 (0.0)	299 (100.0)	14 (5.5)	
2	87 (7.2)	0 (0.0)	0 (0.0)	87 (34.3)	
>= 3	153 (12.6)	0 (0.0)	0 (0.0)	153 (60.2)	
*Total*	*1*,*214 (100*.*0)*	*662 (100*.*0)*	*299 (100*.*0)*	*254 (100*.*0)*	
**Number of agents involved**					<0.001
1	910 (75.0)	535 (80.9)	205 (68.6)	170 (66.9)	
2	190 (15.7)	84 (12.7)	62 (20.7)	44 (17.3)	
>= 3	114 (9.4)	42 (6.4)	32 (10.7)	40 (15.7)	
*Total*	*1*,*214 (100*.*0)*	*661 (100*.*0)*	*299 (100*.*0)*	*254 (100*.*0)*	
**Kind of agents involved**					<0.001
T36-T50 & T51-T65	166 (13.7)	80 (9.8)	51 (12.7)	35 (10.8)	
T36-T50^2^	268 (22.1)	107 (22.9)	76 (31.7)	85 (37.0)	
T51-T65^3^	776 (64.1)	470 (67.3)	172 (55.6)	134 (52.2)	
*Total*	*1*,*210 (100*.*0)*	*657 (100*.*0)*	*299 (100*.*0)*	*254 (100*.*0)*	
**Intentionality**					
Accidental (unintentional)	40 (3.3)	34 (5.1)	6 (2.0)	0 (0.0)	
Substances of abuse	790 (65.1)	555 (84.0)	162 (54.2)	73 (28.7)	
Intentional self-harm	261 (21.5)	67 (10.1)	97 (32.4)	97 (38.2)	
Undetermined intentionality	123 (10.1)	5 (0.8)	34 (11.4)	84 (33.1)	<0.001
*Total*	*1*,*214 (100*.*0)*	*661 (100*.*0)*	*299 (100*.*0)*	*254 (100*.*0)*	
**Monitoring vital parameters**					<0.001
Yes	772 (63.6)	401 (60.7)	227 (75.9)	144 (56.7)	
No	442 (36.4)	260 (39.3)	72 (24.1)	110 (43.3)	
*Total*	*1*,*214 (100*.*0)*	*661 (100*.*0)*	*299 (100*.*0)*	*254 (100*.*0)*	
**Laboratory testing**					<0.001
Yes	703 (57.9)	315 (47.7)	216 (72.2)	172 (67.7)	
No	511 (42.1)	346 (52.3)	83 (27.8)	82 (32.3)	
*Total*	*1*,*214 (100*.*0)*	*661 (100*.*0)*	*299 (100*.*0)*	*254 (100*.*0)*	
**Medical imaging**					<0.001
Yes	276 (22.7)	103 (15.6)	81 (27.1)	92 (36.2)	
No	938 77.3)	558 (84.4)	218 (72.9)	162 (63.8)	
*Total*	*1*,*214 (100*.*0)*	*661 (100*.*0)*	*299 (100*.*0)*	*254 (100*.*0)*	
**Intravenous drip / medication**					
Yes	763 (62.9)	393 (59.5)	220 (73.6)	150 (59.1)	<0.001
No	451 (37.1)	268 (40.5)	79 (26.4)	104 (40.9)	
*Total*	*1*,*214 (100*.*0)*	*661 (100*.*0)*	*299 (100*.*0)*	*254 (100*.*0)*	
**Wound, catheter, ostomy care/ minor surgical intervention**					
Yes	109 (9.0)	57 (8.6)	27 (9.0)	25 (9.8)	0.85
No	1,105 (91.0)	604 (91.4)	272 (91.0)	229 (90.2)	
*Total*	*1*,*214 (100*.*0)*	*661 (100*.*0)*	*299 (100*.*0)*	*254 (100*.*0)*	
**Invasive techniques**					<0.001
Yes	685 (56.4)	257 (38.9)	224 (74.9)	204 (80.3)	
No	529 (43.6)	404 (61.1)	75 (25.1)	50 (19.7)	
*Total*	*1*,*214 (100*.*0)*	*661 (100*.*0)*	*299 (100*.*0)*	*254 (100*.*0)*	
**Patient restraint**					<0.001
Yes	86 (7.1)	27 (4.1)	38 (12.7)	21 (8.3)	
No	1,128 (92.9)	634 (95.9)	261 (87.3)	233 (91.7)	
*Total*	*1*,*214 (100*.*0)*	*661 (100*.*0)*	*299 (100*.*0)*	*254 (100*.*0)*	
**Other treatment**					0.15
Yes	1,167 (96.1)	629 (95.2)	291 (97.3)	247 (97.2)	
No	47 (4.9)	32 (4.8)	8 (2.7)	7 (2.7)	
*Total*	*1*,*214 (100*.*0)*	*661 (100*.*0)*	*299 (100*.*0)*	*254 (100*.*0)*	
**Use of antidotes**					<0.001
Yes	27 (2.2)	5 (0.8)	9 (3.0)	13 (5.1)	
No	1,187 (97.8)	656 (99.2)	290 (97.0)	241 (94.9)	
*Total*	*1*,*214 (100*.*0)*	*661 (100*.*0)*	*299 (100*.*0)*	*254 (100*.*0)*	
**Psychiatric care**					<0.001
No psychiatric consultation	490 (40.4)	405 (61.3)	64 (21.4)	21 (8.3)	
Psychiatric consultation	399 (32.9)	224 (33.9)	136 (45.5)	39 (15.4)	
Admission to psychiatry	288 (23.7)	11 (1.7)	89 (29.8)	188 (74.0)	
Compulsory admission to psychiatry	37 (3.0)	21 (3.2)	10 (3.3)	6 (2.4)	
*Total*	*1*,*214 (100*.*0)*	*661 (100*.*0)*	*299 (100*.*0)*	*254 (100*.*0)*	
**Fate of the patient after discharge hospital**					<0.001
Home	982 (80.9)	558 (84.4)	246 (82.3)	178 (70.1)	
Another, non-university hospital	52 (4.3)	5 (0.8)	12 (4.0)	35 (13.8)	
Psychiatric hospital	107 (8.8)	39 (5.9)	33 (11.0)	35 (13.8)	
Home for the elderly	1 (0.1)	1 (0.2)	0 (0.0)	0 (0.0)	
Deceased	1 (0.1)	0 (0.0)	0 (0.0)	1 (0.4)	
Other	17 (1.4)	6 (0.9)	7 (2.3)	4 (1.6)	
Unknown	54 (4.4)	52 (7.9)	1 (0.3)	1 (0.4)	
*Total*	*1*,*214 (100*.*0)*	*661 (100*.*0)*	*299 (100*.*0)*	*254 (100*.*0)*	

^1^ Chi-square and Fisher’s exact test

^2^ T36-T50: Drugs, medicaments and biological substances

^3^ T51-T65: Substances chiefly nonmedicinal as to source

**Table 3:** Agents used by patients admitted for poisoning to the emergency department of the Ghent University Hospital in 2017, classified by all agents, single or combined use, and by gender.

ICD-10 Agents^1^		Total	1 agent	>1 agent	Male	Female	p-value^2^
		n = 1,701	n = 910	n = 791	n = 1,024	n = 677	
**T51**	**Alcohol**	**901 (53.0)**	**730 (80.2)**	**171 (21.6)**	**597 (58.3)**	**304 (44.9)**	
T51.0	Ethanol	899 (52.9)	729 (80.1)	170 (21.5)	596 (58.2)	303 (44.8)	<0.001
**T40**	**Narcotics and psychodysleptics (hallucinogens)**	**229 (13.5)**	**47 (5.2)**	**182 (23.0)**	**176 (17.2)**	**53 (7.8)**	
T40.2	Other opioids	30 (1.8)	6 (0.7)	24 (3.0)	17 (1.7)	13 (1.9)	0.632
T40.5	Cocaine	83 (4.9)	17 (1.9)	66 (8.3)	63 (6.2)	20 (3.0)	0.008
T40.7	Cannabis (derivatives)	79 (4.6)	15 (1.6)	64 (8.1)	65 (6.3)	14 (2.1)	<0.001
T40.1	Heroin	14 (0.8)	5 (0.5)	9 (1.1)	13 (1.3)	1 (0.1)	
**T43**	**Psychotropic drugs, NEC**^3^	**199 (11.7)**	**39 (4.3)**	**160 (20.2)**	**99 (9.7)**	**100 (14.8)**	
	Antidepressants	78 (4.6)	13 (1.4)	65 (8.2)	21 (2.1)	57 (8.4)	<0.001
	Antipyschotics	43 (2.5)	10 (1.1)	33 (4.2)	17 (1.7)	26 (3.8)	0.003
	Psychostimulants	78 (4.6)	16 (1.8)	62 (7.8)	61 (6.0)	17 (2.5)	0.006
**T42**	**Anti-epileptic, sedative-hypnotic, antiparkinsonism drugs**	**179 (10.5)**	**23 (2.5)**	**156 (19.7)**	**77 (7.5)**	**102 (15.1)**	
T42.4	Benzodiazepines	165 (9.7)	21 (2.3)	144 (18.2)	68 (6.6)	97 (14.3)	<0.001
	Anti-epileptics	5 (0.3)	1 (0.1)	4 (0.5)	3 (0.3)	2 (0.3)	
**T39**	**Nonopioid analgesics, antipyretics, antirheumatics**	**70 (4.1)**	**21 (2.3)**	**49 (6.2)**	**13 (1.3)**	**57 (8.4)**	
T39.1	Paracetamol	42 (2.5)	14 (1.5)	28 (3.5)	11 (11)	31 (4.6)	<0.001
T39.3	Other nonsteroidal anti-inflammatory drugs [NSAIDs]	27 (1.6)	6 (0.7)	21 (2.7)	2 (0.2)	25 (3.7)	<0.001
**T46**	**Agents primarily affecting the cardiovascular system**	**28 (1.6)**	**1 (0.1)**	**27 (3.4)**	**9 (0.9)**	**19 (2.8)**	
	Beta-blockers	11 (0.6)	0 (0.0)	11 (1.4)	0 (0.2)	9 (1.3)	
**T58**	**Carbon monoxide**	**20 (1.2)**	**17 (1.9)**	**3 (0.4)**	**15 (1.5)**	**5 (0.7)**	**0.234**
**T59**	**Other gases, fumes and vapours**	**13 (0.8)**	**12 (1.3)**	**1 (0.1)**	**8 (0.8)**	**5 (0.7)**	
**T41**	**Anaesthetics and therapeutic gases**	**11 (0.6)**	**2 (0.2)**	**9 (1.1)**	**9 (0.9)**	**2 (0.3)**	
**T47**	**Agents primarily affecting the gastrointestinal system**	**9 (0.5)**	**0 (0.0)**	**9 (1.1)**	**3 (0.3)**	**6 (0.9)**	
**T54**	**Corrosive substances**	**7 (0.4)**	**5 (0.5)**	**2 (0.3)**	**4 (0.4)**	**3 (0.4)**	
**T45**	**Primarily systemic and haematological agents, NEC**	**7 (0.4)**	**2 (0.2)**	**5 (0.6)**	**2 (0.2)**	**5 (0.7)**	
**T700**^3^	**Other**^4^	**6 (0.4)**	**4 (0.4)**	**2 (0.3)**	**2 (0.2)**	**4 (0.6)**	
**T36**	**Systemic antibiotics**	**3 (0.2)**	**0 (0.0)**	**3 (0.4)**	**2 (0.2)**	**1 (0.1)**	
**T38**	**Hormones and their synthetic substitutes and antagonists, NEC**	**4 (0.2)**	**0 (0.0)**	**4 (0.5)**	**1 (0.1)**	**3 (0.4)**	
**T50**	**Diuretics and unspecified drugs, medicaments and biological substances**	**4 (0.2)**	**1 (0.1)**	**3 (0.4)**	**3 (0.3)**	**1 (0.1)**	
**T55**	**Soaps and detergents**	**1 (0.1)**	**1 (0.1)**	**0 (0.0)**	**0 (0.0)**	**1 (0.1)**	
**T65**	**Other and unspecified substances**	**2 (0.1)**	**1 (0.1)**	**1 (0.1)**	**0 (0.0)**	**2 (0.3)**	
**T49**	**Agents primarily affecting skin, mucous membrane and ophthalmological, otorhinolaryngological and dental drugs**	**1 (0.1)**	**1 (0.1)**	**0 (0.0)**	**0 (0.0)**	**1 (0.1)**	
**T60**	**Pesticides**	**1 (0.1)**	**1 (0.1)**	**0 (0.0)**	**0 (0.0)**	**1 (0.1)**	
**T52**	**Organic solvents**	**2 (0.1)**	**0 (0.0)**	**2 (0.3)**	**2 (0.2)**	**0 (0.0)**	
**T44**	**Drugs primarily affecting the autonomic nervous system**	**2 (0.1)**	**1 (0.1)**	**1 (0.1)**	**1 (0.1)**	**1 (0.1)**	
**T37**	**Other systemic anti-infectives and antiparasitics**	**1 (0.1)**	**0 (0.0)**	**1 (0.1)**	**1 (0.1)**	**0 (0.0)**	
**T56**	**Metals**	**1 (0.1)**	**1 (0.1)**	**0 (0.0)**	**0 (0.0)**	**1 (0.1)**	
		**1,701 (100.0)**	**910 (100.0)**	**791 (100.0)**	**1,024 (100)**	**677 (100.0)**	

^1^ The main ICD-10-groups are listed and the most important agents of those main groups.

^2^ Chi-square and Fisher’s exact test

^3^ NEC = Not Elsewhere Classified

^4^ Other: pushpin, absorbent granules, tinplate, toothbrush, lighter, plasticine.

For the multilevel analysis of the factors associated with the type of hospitalization, the group of patients who were admitted only once were considered as well as the group of patients who were admitted more than once.

### Data collection

We used the minimum-hospital-data (MZG) as a first source of information. This obligatory registration system contains administrative, medical and nursing data of hospitalized patients such as diagnoses, treatments provided, intentionality and discharge status. For patients hospitalized, the ICD-10 codes were used, available in the section “diagnosis” of the minimum-hospital-data (MZG). The before last digit of the ICD-10-codes gives an indication of the intentionality.

The emergency registration system for hospital EDs, named UREG, was used as the second source. According to a Royal Decree, each Belgian hospital with a specialized ED has to register administrative and medical data on all ED patients and to transmit them to the Federal Public Service Health (FPS Health). UREG provides demographic data (age, gender, marital status, nationality, type of insurance), admission and discharge times, location prior to admission and type of transport to the hospital. Data about the reason for admission, symptoms, type of agent(s), diagnosis, degree of severity, type of discharge, destination after discharge, were also collected. This registration system also provides data on intentionality by mentioning either intake of a substance of abuse, suicide and/or self-harm.

The third source of information was the electronic patient file of the patient (EPD), available for both ambulatory and hospitalized patients. We used data such as the Glasgow Coma Score (GCS), intentionality, agents involved and consultations for psychiatric care. Data on intentionality from MZG and UREG, were verified in the EPD. If the intentionality was not clear from data of the different sources, the case was categorized as ‘undetermined’.

Financial data on direct medical costs were obtained from the hospital’s financial department. They were abstracted from the invoices of the individual patients and expressed in EUR (1 EUR = 1.17 USD, December 2017). Cost was defined as the payer’s cost. In case of an admission to the hospital, the payer is (1) on the one hand the government, through contributions from the health and disability insurance, obligatory for people in Belgium, and (2) on the other hand the individual patient. The financing of Belgian hospitals is complex. A part of the hospital budget is fixed and is paid monthly to the hospitals (system of budgetary twelfths) by the government via seven Belgian insurance institutions. Another part is variable and consists of an amount per admission and per hospitalization day. This variable cost is charged by the hospital to two parties: a major part is paid by the government via the seven insurance institutions, a smaller part is paid by the individual patient via the hospital’s invoice to the patient (usually between 18 and 20% of the variable cost). The invoice contains four parts: accommodation and nursing, physicians’ fees, use of pharmaceuticals and other costs (e.g. bottle of drinking water, use of refrigerator and/or television). The payer’s hospital cost presented in this study is the cost paid by the government (fixed and variable part, paid via the insurance companies) plus the cost paid by the individual patient. Cost is calculated on the patients with an obligatory insurance.

### Statistical analysis

A descriptive study was performed on the variables using Pearson Chi-Square test and Fisher’s Exact Test to compare categorical data between groups.

A multilevel multinomial logistic regression with generalized logit link function was used to analyse the factors associated with the type of hospitalization. Univariate analysis was used calculating the unadjusted odds ratios to assess the predicting variables related to the hospital admission type.

In the multivariate analysis, the step-by-step method was used with the variables which in the univariate analysis achieved a statistically significant association (p<0.05) or had a clear clinical and/or biological significance. The predictors of the final model were selected based on the Akaike Information Criterion (AIC). The discriminatory power of the model was assessed through the determination of the area under the ROC curve (AUC). To avoid overoptimistic areas under the ROC curve and to validate the model, k-fold cross-validation (k = 10) was applied. A multilevel multinomial logistic regression was applied on a dataset containing one record per patient. The sample of data was partitioned at random into 10 complementary subsets. For each subset, the predicted probabilities were estimated on the sample data excluding that particular subset. All analyses were performed using SPSS 25.0 (IBM^®^).

### Ethical considerations

The study protocol was approved by the Ethical Committee of the Ghent University Hospital (approval number B670201732651).

## Results

### Demographics and characteristics of the patients on admission

In total, 1,214/34,000 (3.6%) admissions were included, of whom 62.2% were male (Table 1). Of these admissions, 54.5% received ambulatory care, 24.6% had to stay for 24-hours (or less) in the ED, 20.9% were hospitalized or admitted to the intensive care unit (ICU). Mean age was 37 years (SD 15.56y), with 43.0% between 21-40y and the age group >60y being less represented. Of all patients, 90.9% was admitted once, 5.8% twice and 3.4% three times or more.

The majority was unmarried, widow(er) or divorced and 1,175 had a Belgian obligatory insurance. Forty-nine percent presented on Fridays or during the weekend. Sixty percent came from home and 26.5% from a public place. In the group of hospitalized patients, 80.3% came from home, 12.2% from a public place and 40.2% were transported by ambulance.

### Characteristics of hospitalized patients, examinations, treatment and follow-up

As shown in Table 2, 21.2% of the patients showed changes in consciousness and 16.8% behavioural and emotional disorders. The number of patients with a GCS score lower than 15 was higher (24.3% versus 21.2%) than the number of patients with changes in consciousness. This may be explained by the fact that consciousness was registered as a UREG-parameter by the nurse during the admission process. The GCS, which is more accurate, was noted by the doctor in a later stage in the electronic file (EPD) of the patient. Some patients may have evolved to a lower level of consciousness. However, we should also keep in mind that a decrease in consciousness is not a rigourous, but subjective interpretation.

According to the Manchester Triage Scale, 11.8% were evaluated as not urgent but were nevertheless hospitalized. The mean length of hospital stay was 1.12 days (SD 3.12) and the median length was 0.00 days (IQR 0.00–1.00). Subtracting the 661 ambulatory patients who stayed less than 1 day (the ED-amb patients), we obtained a mean of 2.46 (SD 4.26) and a median of 1.00 (IQR 1.00–3.00) days for the remaining 553 patients. N-acetylcysteine was administered in 3.4% and naloxone in 0.8% of admissions assessed as intentional self-harm. Thiamine was given to 32.1% of admissions involving ethanol. Psychiatric consultations were performed in 59.6% of all admissions and in 95.0% of admissions for intentional self-harm. Most patients (80.9%) could return home after discharge from the hospital and 8.8% were referred to a psychiatric hospital. One patient died in the intensive care unit (mortality of 0.1%). Monitoring of vital parameters and administration of medication and/or an intravenous drip were the most common treatments.

### Agents involved

A total number of 1,701 agents were involved (Table 3). Substances most commonly involved were ethanol (52.9%), benzodiazepines (9.7%), cocaine (4.9%), cannabis (4.6%), antidepressants (4.6%) and psychostimulants (4.6%).

In 75.0% of admissions only one agent was taken. Most popular combinations were ethanol with benzodiazepines (36 admissions), ethanol with cannabis (24), ethanol with cocaine (18), benzodiazepines with antidepressants (14) and ethanol with amphetamines (13 admissions).

Table 3 gives an overview of the agents used by men and women separately. Women used more frequently benzodiazepines, antidepressants, paracetamol and NSAIDs, while men used more ethanol, cocaine, cannabis, psychostimulants, heroin and anaesthetics (ketamine and procaine).

### Factors associated with hospitalization type

Table 4 shows the results of the univariate (unadjusted OR) and multivariate (adjusted OR) analysis performed to identify the factors associated with hospitalization type using the ED-amb population as the reference. In the univariate analysis, the odds ratios of the following variables were calculated to assess the predicting variables related to the hospital admission type: gender, age, marital status, residence, day of the week of admission, time of admission, victim location, referral, transport, route of exposure, degree of severity, Glasgow Coma Score, number of agents involved, type of agents involved, use of antidotes, involvement of ethanol, antidepressants, antipsychotics, psychostimulants, benzodiazepines, cocaine, cannabis, paracetamol and NSAIDs. In the final model (multivariate analysis), age, time of admission, victim location, degree of severity, use of antidotes, involvement of ethanol, antidepressants, antipsychotics, psychostimulants and benzodiazepines were associated with the hospitalization type. After cross-validation, the estimated AUCs were 81.3% (95%CI: 78.7%-83.8%) for ED-amb, 78.4% (95%CI: 75.5%-81.3%) for ED-24h and 80.2% (95%CI: 77.4%-83.1%) for Hosp.

**Table 4:** Univariate and multivariate analysis of factors associated with hospitalization type of patients admitted for poisoning to the Ghent University Hospital in 2017.

	ED-24h (ref: ED-amb)		Hosp (ref: ED-amb)	
	UNADJUSTED	ADJUSTED	UNADJUSTED	ADJUSTED
	OR^1^ (CI^2^)	OR^1^ (CI^2^)	OR^1^ (CI^2^)	OR^1^ (CI^2^)
**Age**				
>60y	5.29 (2.82–9.90)*	3.58 (1.74–7.33)*	7.08 (3.27–15.30)*	5.13 (2.12–12.41)*
41-60y	4.22 (2.64–6.74)*	2.56 (1.48–4.44)*	7.55 (4.15–13.72)*	4.78 (2.37–9.64)*
21-40y	2.31 (1.47–3.65)*	1.79 (1.07–3.02)*	4.05 (2.25–7.28)*	2.80 (1.43–5.49)*
14-20y	REF	REF	REF	REF
**Hour of admission**				
8am-12am	3.31 (1.53–7.12)*	2.77 (1.23–6.24)*	6.41 (2.98–13.81)*	5.17 (2.20–12.17)*
12am-4pm	5.40 (2.75–10.61)*	3.72 (1.79–7.74)*	8.73 (4.30–17.73)*	6.01 (2.69–13.39)*
4pm-8pm	7.36 (3.91–13.85)*	5.28 (2.65–10.52)*	6.33 (3.16–12.67)*	4.50 (2.04–9.94)*
8pm-12pm	8.94 (4.80–16.65)*	6.99 (3.58–13.64)*	5.73 (2.87–11.45)*	4.95 (2.27–10.77)*
12pm-4am	0.85 (0.42–1.72)	0.82 (0.39–1.72)	0.98 (0.46–2.07)	1.03 (0.45–2.36)
4am-8am	REF	REF	REF	REF
**Victim location**				
Other	0.87 (0.59–1.29)	1.90 (1.09–3.32)*	0.32 (0.18–0.54)*	0.86 (0.41–1.79)
Public place	0.32 (0.22–0.46)*	1.75 (1.15–2.67)*	0.21 (0.13–0.31)*	2.39 (1.46–3.93)*
Home	REF	REF	REF	REF
**Manchester triage score**				
Urgent or very urgent	3.16 (1.92–5.19)*	2.67 (1.54–4.64)*	4.37 (2.60–7.34)*	3.87 (2.13–7.03)*
Less urgent	2.06 (1.37–3.10)*	1.96 (1.26–3.06)*	1.80 (1.15–2.83)*	1.78 (1.08–2.93)*
Not urgent	REF	REF	REF	REF
**Use of antidotes**				
Yes	4.05 (1.32–12.44)*	7.35 (2.12–25.47)**	7.12 (2.41–21.02)*	13.07 (3.80–44.90)*
No	REF	REF	REF	REF
**Antidepressants**				
Yes	3.20 (1.63–6.27)*	2.40 (1.09–5.26)*	5.63 (2.94–10.82)*	4.10 (1.85–9.08)*
No	REF	REF	REF	REF
**Antipsychotics**				
Yes	2.44 (1.08–5.51)*	1.33 (0.52–3.36)	3.96 (1.78–8.81)*	2.06 (0.80–5.29)
No	REF	REF	REF	REF
**Psychostimulants**				
Yes	0.84 (0.44–1.59)	1.62 (0.75–3.50)	1.50 (0.83–2.72)	2.72 (1.28–5.81)*
No	REF	REF	REF	REF
**Benzodiazepines**				
Yes	3.54 (2.27–5.52)*	2.59 (1.56–4.31)*	3.44 (2.14–5.55)*	1.92 (1.10–3.37)*
No	REF	REF	REF	REF
**Ethanol**				
Yes	0.72 (0.52–1.00)*	1.68 (1.08–2.61)*	0.55 (0.44–0.76)*	1.52 (0.94–2.47)
No	REF	REF	REF	REF

^1^ OR = Odds Ratio

^2^ CI = Confidence Interval

* significant, p<0.05

REF = Reference category

The estimated odds for ED-24h or Hosp versus ED-amb increased with age. The estimated odds for ED-24h and Hosp versus ED-amb were much higher for day and evening hours between 8am and 12pm than for night hours between 12pm and 8am. Patients triaged as urgent or very urgent by the Manchester triage scores were associated with higher estimated odds of being admitted to ED-24h or Hosp. Needing antidotes and involvement of antidepressants and benzodiazepines were also factors associated with a greater risk of ED-24h or Hosp as opposed to ED-amb. Ethanol was significantly associated with a greater risk of ED-24h and psychostimulants with a greater risk of Hosp.

### Cost

Table 5 shows the total, mean and median costs for the 1,175/1,214 admissions (including readmissions) for patients with an obligatory insurance. Total direct costs were €1,512,346: €125,326 for 637 ED-amb patients, €389,539 for 290 ED-24h patients and €997,481 for 248 Hosp patients. The total cost for the initial care in the ambulatory ward of the ED accounted for €198,677 (with inclusion of the cost for the ED-amb patients, the ED-24h patients and the Hosp patients). The total cost for ED-amb patients who were discharged home after their care was €125,326 or a mean of €197 per patient. The total cost for both ED-24h patients and Hosp patients (with inclusion of the cost of the initial care in the ED-amb ward) was €1,387,020 representing a mean cost of €2,578 per patient.

**Table 5:** Total, mean and median cost in EUROs of patients admitted for poisoning to the Ghent University Hospital, 2017.

**Type of hospitalisation**^1^	**Total cost**^5^**, €**	**Cost in the ED unit**	**Cost in the hospitalisation unit**
Total (all types of hospitalisation)	**1,512,346**	198,677	1,313,669
ED-amb^2^	**125,326**	125,326	0.0
ED-24h^3^	**389,539**	38,383	351,156
Hosp^4^	**997,481**	34,968	962,513
**Type of hospitalisation**^1^	**Mean cost**^6^**, € (SD)**	**Cost in the ED unit**	**Cost in the hospitalisation unit**
Total (all types of hospitalisation)	**1,287 (2,653)**	169	1,118
ED-amb^2^	**197(147)**	197	0.0
ED-24h^3^	**1,343 (292)**	132	1,211
Hosp^4^	**4,022 (4,766)**	141	3,881
**Type of hospitalisation**^1^	**Median cost**^7^**, € (Q1-Q3)**	**Cost in the ED unit**	**Cost in the hospitalisation unit**
ED-amb^2^	**423 (154–1,472)**	140 (82–216)	0.1 (0.1–0.1)
ED-24h^3^	**164 (93–253)**	164 (93–253)	0.0 (0–0)
Hosp^4^	**1,301 (1,237–1,396)**	118 (72–173)	1,170 (1,109–1,261)
Hosp	**2,854 (2,157–3,768)**	117 (60–189)	2,745 (2,024–3,5878)

^1^ Costs are categorised into type of admission and are presented in EUROs

^2^ ED-amb: ambulatory patients discharged home after treatment in the emergency department

^3^ ED-24h: patients requiring 24-hours-observation at maximum in the emergency department

^4^ Hosp: patients admitted to a hospital ward/ intensive care unit

^5^ Total cost, categorised by emergency department (ED) cost and hospitalisation cost

^6^ Mean cost, categorised by emergency department (ED) and hospitalisation cost; SD = standard deviation

^7^ Median cost, categorised by emergency department (ED) cost and hospitalisation cost; Q1-Q3 = Interquartile range.

The age groups 15-20y, 21-40y, 41-60y and the group older than 60y represented 17.9%, 42.1%, 32.2% and 7.7% of the total study group and accounted for 8.6%, 37.8% and 44.0% and 9.6% of total costs respectively.

The mean cost per admission amounting to €1,287 (SD 2,653), was covered by the government for 95.7% via the obligatory insurance and for 4.3% by the fee for the patient. The median cost was €423 (IQR €154–1,471).

When excluding the readmissions, the mean cost for the 1,042 patients was €1,264 (SD 2,692), of which 95.5% was covered by the government via the obligatory insurance and 4.5% by a fee to the patient. The mean cost was €199 for ED-amb patients, €1,359 for ED-24h patients and €4,146 for hospitalized patients. The median cost was €372 (IQR €152–1,440).

In cases of ethanol poisoning (whether or not in combination with other agents, the mean cost per admission was €1,216 (SD €2,691) for the 1,175 admissions (including readmissions), with a median cost of €376 (IQR 154–1,389). In cases of poisoning without involvement of ethanol, the mean cost per admission was €1,490 (SD 2,533), with a median cost of €376 (IQR €154–2,036).

When medicinal agents were involved (T36-T50, poisoning by drugs, medicaments and biological substances), the median cost for hospitalised poisoned patients (including readmissions) was €1,983 (IQR €1,310–2,036). When non-medicinal agents were involved (T51-T65, toxic effects of substances chiefly nonmedicinal as to source), the median cost was €1,534 (IQR €1,272–2,782).

## Discussion

This study analyzed data of 2017 of all poisoning cases in a university hospital, with analysis of the factors associated with hospitalization type and cost calculation based on the individual invoices. As far as we know, this study is the first to combine these aspects, which may prove to be valuable for healthcare professionals and policy makers.

Patients with acute poisoning represented 3.6% of total patient population. Figures from other countries are lower (range 0.3–1.7%) [3,6,11,12,18]. Verstraete & Buylaert [32], who analyzed poisonings between 1983 and 1990 in the same hospital, but with exclusion of single poisonings with ethanol, reported 3.2%.

The mean age average of 37 years in our study was within the range of most studies (33-40y) [3,4,10,11,14,17,18,32] although some studies found a lower mean age (range 23-28y) [2,5,12].

Men accounted for 62.2% of the admissions, compared to 44.0% [32] between 1983–1990. This can be due to the inclusion in our study of single poisonings with only ethanol, as men accounted for 66.3% of these cases.

In our study, patients were most likely to consult the ED between 8pm and 12 pm and between 12 pm and 4 am (22.4%). Other healthcare services are often not available at these times and psychosocial problems are then probably more prevalent. The higher consumption of ethanol at these hours is possibly another contributing factor.

The socially accepted drug ethanol was used in 52.9%: in 80.1% of the admissions as a single agent and in 21.5% as concomitant substance. In a recent study of Muňoz et al.[18] in Spain, ethanol was involved in 44.7% of cases, which is close to our result (52.9%).

In the Netherlands, Duineveld et al. [4] analysed acute intoxications in six hospitals. They reported the use of ethanol whether or not in combination with other drugs in 318/1,183 patients. In cases of drugs of abuse (DOA), ethanol was involved in 73.5%, of which 60.7% mono-intoxications and 39.3% in combination with (illicit) drugs. In the 735/1.183 suicide attempts in the study of Duineveld et al. [4], seven cases of mono-intoxications with ethanol with the intention of self-harm were recorded. It is not clear if ethanol was involved in other cases of intentional self-harm which may to some extent explain the low percentage of cases with ethanol involved.

The percentages of ethanol mentioned in the studies on poisoning in Oslo hospitals of Hovda [14] and Lund [17] (17% and 18% respectively) are lower than in our study, but one should keep in mind that there is one ambulance service and a large outpatient clinic and four public emergency hospitals in Oslo. The majority of ethanol poisonings are referred to the outpatient clinic [37,38]. In the hospitals, pharmaceuticals are most frequently involved. Data from the hospitals and the outpatient clinic must be seen together.

The ten most frequently used agents are comparable in most studies, although their ranking could varies [3,4,14,18,20,25].

In our study, benzodiazepines were mentioned in 9.7%., cocaine was used in 4.9% and psychostimulants in 4.6% In the study of Duineveld et al. [4], cocaine was involved in 27.3% of drugs of abuse cases and psychostimulants in 21.0%. The more liberal drug policy in the Netherlands may be one of the factors explaining this higher percentage.

Carbon monoxide was involved in 1.2%, versus in 11.7% during the period 1983–1990 in our hospital [32]. A possible explanation may be found in the regulatory measures on technical appliances by the government. Antidotes were given in 2.2% of admissions, with naloxone in 0.5% and N-acetylcysteine in 1.1%. In Oslo [17], naloxone was given in 17% and N-acetylcysteine in 11%.

The overall percentage of patients receiving psychiatric care was high, presumably because of the psychiatric nature of many poisoning admissions and the 24/7 availability of a psychiatrist in GUHED. For intentional poisonings, it amounted to even 95.0% compared with 67.0% and 90.0% in the studies of Lund [17] and Hendrix [20]. Providing psychiatric help with a low threshold is in accordance with the current National Institute for Health and Care Excellence (NICE) guidelines [39], as intentional self-harm in the past is the strongest known predictor of a later successful suicide attempt.

Following the care in the ED, 54.4% patients were discharged home and 24.6% left the ED within 24 hours. Only 20.9% of patients were hospitalized. In the earlier study from GUHED, Verstraete & Buylaert [32] reported 27.8% of patients being discharged home from ED. The exclusion of ethanol cases when it was the sole agent in this previous study probably explains this difference in the discharge rate.

With regard to the factors influencing the hospitalization type, we found that, among other criteria, antidepressants and benzodiazepines were significantly associated with a higher estimated odds for ED-24h or Hosp versus ED-amb. It is also not surprising that the odds for ED-24h or Hosp versus ED-amb is higher for urgent or very urgent Manchester Triage Scores than for patients with a non or less urgent scores. The need for antidotes is also a factor more frequently leading to hospitalization. The higher odds for ED-24h and Hosp during the day and evening hours compared with night hours was unexpected and requires further investigation.

When analyzing the cost, we found a mean cost of €1,287 per admission in our study: a mean of €197 per ED-amb patient and €1,118 hospitalisation cost for ED-24h patients and Hosp patients. In Spain, Muňoz et al. [18] calculated a mean cost of €571 (indexed 2017: €586): €222 (indexed 2017: €228) per ED-amb patient and €4,121 (indexed 2017: €4,224) for hospitalised patients (both ED-patients who stayed longer than 6 hours and hospitalized patients). Compared with our total mean cost of €1,287, the cost calculated by Muňoz (€586) is lower. This may be due to the fact that our study comprises 54.2% of ambulatory patients and 45.8% of hospitalized patients versus 11.02% (359/3,159) ambulatory patients and 88.8% (2,836/3,195) hospitalized patients in the study of Muňoz. The cost for ED-amb patients in our study (€197) is indeed comparable to that in the study of Muňoz (€228) but is lower for hospitalized patients (€2,578 versus €4,224) which is difficult to explain. We suppose that, as more patients were ambulatory in Spain, the admission protocol to observe patients during more than 6 hours in the ED or to hospitalize them, was more selective than in our study. This could have led to a higher degree of severity in the Muňoz paper. It should however be mentioned that this hypothesis is not supported by the mean duration of the hospital stay. The mean hospital stay in the study in Spain is indeed very similar to ours (1.19 versus 1.12 days), with a mean stay for ED-24h and hospitalized patients that is even shorter in the Spain study (1.99 days) than in ours (2.46 days). Another hypothesis is that the type of poisonings included in the study of Muñoz is different from our study, as it is based on the information provided by the diagnosis-related groups (DRG), which could not be used in our study.

In our study, the median cost for hospitalized patients with medicinal agents involved (T36-T50, poisoning by drugs, medicaments and biological substances,) was €1,983. Okumara et al. [25] reported a median cost of $1,776 (indexed 2017: $2,134 or €1,824) for inpatients with drug poisoning (ICD-10, T36-T50,) which is close to our figure (€1,983). Okumara et al. also reported that the age group between 20-39y (19,200/37,200 patients, i.e. 51%) was responsible for 50% of the costs, which is in the same range of our results: the group 21-40y (495/1,175 or 42.1%) was responsible for 37.8% of the costs.

The costs in two studies available for Belgium [19,20], are comparable with our figures. Hendrix [20] calculated €828/patient (indexed January 2017: € 948.48) from admission until ED-discharge for deliberate self-poisoning cases (substances of abuse and intentional self-harm, excluding alcohol as single drug). The mean cost/admission in our study using the same inclusion criteria was in the same order of magnitude, i.e. €796 (SD 2,340). Verelst [19] mentioned an estimated ED-cost for ethanol poisoning of €541/patient (indexed January 2017: €620). In our study, the mean ED-cost for alcohol poisoning (ED-amb and ED-24h) was in the same order of magnitude: €418/patient (SD 470).

The total consolidated Public Health Expenditure of the National Health System in Belgium in 2017 was US$ 4,774 (€ 4,224) per capita [40–42]. This represents US$ 53.9 billion (€ 47.7 billion) or 10% of the Gross Domestic Product (GDP). In 2015, Belgian hospitals accounted for nearly 33% (US$ 17.7 billion or € 15.6 billion) of health spending (versus 40% in Organisation for Economic Cooperation and Development (OECD) countries on average). In Ghent University Hospital, there were 575,000 hospital admissions in 2017 of which 1,214 (0.21%) for poisoning.

### Strengths and weaknesses

As the present study was carried out in one university hospital, data cannot simply be extrapolated to other settings. It would be of interest to use the same methodology in other hospitals of other levels and with different settings. Because of the retrospective character and the fact that we had to rely on data collected by doctors, nurses and/or administrative staff during their routine work, it is likely that some admissions and information is missing in our database and/or that a number of cases were not correctly categorized (e.g. intentionality). Another limitation is that comorbidities were not registered in our study, which obviously may have had an impact on the type of hospitalization and on the duration of the stay in the hospital. Our study found multiple associations with hospitalization type, but obviously we should keep in mind that causal relationship cannot be derived from our data.

A strength of our study is that we used the WHO International Classification of Diseases 10th Revision (ICD-10). This use of a clear and international standard may be a first step in the development of a template for uniform data reporting and comparison between centers in order to facilitate international comparison.

## Conclusion

Acute poisonings account for a considerable proportion of ED admissions representing a significant organizational and financial burden to hospitals and healthcare workers. We observe a high proportion of ethanol poisoning in our study which is of major concern. Our data may provide an incentive for the government to take the necessary preventive measures such as limiting availability by restricting points of sale, set strict age limits for purchase and consumption, increase the price via taxes and forbidding advertising which are proven to be effective [43].

It is difficult to compare results on admissions for poisoning between different EDs. This is due to incompleteness of data on the one hand and the lack of uniformity in reporting on the other hand. A possible solution would be to recommend for epidemiological study purposes a uniform template aimed to report data on poisoning in a standardized way. This is in analogy with registration methods that appeared useful in other domains of emergency medicine like the Utstein template in patients with cardiopulmonary arrest [44] and the registration by the ‘Deutsche Gemeinschaft für Unfallchirurgie’ [45] of patients with severe trauma. Such registrations allow benchmarking of the care. With regard to poisoning cases admitted to the emergency department, a template would be very helpful, with a clear definition of the collected variables using a uniform definition of poisoning, involved agents, intentionality, charges versus cost together with information on country-specific health organisational structure which would be very valuable.

## Supporting information

S1 Table Distribution of patients according to their number of admissions for poisoning in Ghent University Hospital in 2017. (XLSX)

S2 Table Demographic data and characteristics of patients admitted for poisoning according to hospitalization type. Comparison of 1,214 cases including readmissions versus 1,042 patients excluding readmissions. Ghent University Hospital, 2017.
(XLSX)

S3 Table Characteristics, examinations and treatment of patients admitted for poisoning according to hospitalization type. Comparison of 1,214 admissions including readmissions versus 1,042 admissions excluding readmissions, Ghent University Hospital, 2017.
(XLSX)

S4 Table Agents used by patients admitted for poisoning to the emergency department, classified by all agents, single or combined use. Comparison of 1,214 admissions including readmissions versus 1,042 admissions excluding readmissions. Ghent University Hospital, 2017.
(XLSX)

S5 Table Comparison of the unadjusted ORs of 1,214 admissions for poisoning including readmissions with 1,042 admissions excluding readmissions. (XLSX)

S1 File Supporting information file. (ZSAV)

## Abbreviations

ED: Emergency Department
ED-24h: ED-24-hours-observation unit
ED-amb: ED- ambulatory care
EPD: Electronic Patient File
FPS Health: Federal Public Service Health
GUH: Ghent University Hospital
GUHED: Ghent University Hospital Emergency Department
Hosp: Hospitalization unit
ICU: Intensive Care Unit
MICU: mobile intensive care unit
MZG: Minimal hospital data (Minimale Ziekenhuisgegevens)
NICE: National Institute for Health and Care Excellence
NSAIDs: non-steroidal anti-inflammatory drugs
OECD: Organisation for Economic Cooperation and Development
RIZIV: National Health and Disability Insurance Service (Rijksdienst voor Ziekte- en Invaliditeitsverzekering)
UREG: Official Emergency Registration System at Emergency Departments (Urgentie registratiesysteem)
